# Supplementary material for: Ipilimumab, Pembrolizumab, or Nivolumab in Combination with BBI608 in Patients with Advanced Cancers Treated at MD Anderson Cancer Center
Source: Cancers (Basel). 2022 Mar 4;14(5):1330. doi: 10.3390/cancers14051330 (PMC8909492; doi:10.3390/cancers14051330)
Supplement: Supplementary file 1 [file cancers-14-01330-s001.zip › cancers-1596800-supplementary.pdf]

Supplemental Information

Study design

The RP2D for BBI608 in combination with an immunotherapeutic was determined based on the criteria for dose-limiting toxicity (DLT), criteria for determining the maximum tolerated dose (MTD), as well as on available pharmacokinetic, pharmacodynamic data. The DLT evaluation period was six weeks (42 days), starting on the first day of protocol therapy administration. To be considered evaluable for DLT, compliance with the assigned dose of BBI608 must be ≥ 75%.

Initially, 6 patients will be enrolled into each combination arm at a BBI608 dose level of 240 mg twice daily (480 mg total daily dose). Dose-escalation proceeded according to the criteria for dose escalation and criteria for determination of DLT, up to a maximum BBI608 dose-level of 480 mg twice daily (960 mg total daily dose). A dose-level was eligible for consideration as RP2D provided ≤ 1 of 6 patients enrolled experiences DLT at that dose-level. Dose-escalation proceeded independently in each study arm.

Once RP2D was determined for BBI608 in combination with a given immunotherapeutic agent, an additional 15 to 20 evaluable patients was to be enrolled in that arm.

Supplemental Tables

Table S1. Dose modification of BBI608.

**Dose Level**	**BBI608,** mg twice daily
**I**	**240**
Modification Level 1	80
Modification Level 2	80 once daily
**II**	**480**
Modification Level 1	240
Modification Level 2	80
Modification Level 3	80 once daily

Table S2. Molecular profile of 12 patients who underwent treatment with BBI608 and immunotherapy

Pt. ID	Molecular alterations	PDL1		MSI	TMB (m/MB)
		Tumor cell	Tumor-associated immune cell		
1	KRAS G12D, PIK3CA E542K, APC Q1429*, W699*, Y935fs*19, RB1 loss exons 3-17	0%	0%	stable	10
2	MYC amplification – equivocal, BAP1 splice site 123-1G>A, splice site 2057-32_2057-, 1>CCTAGTGGA, truncation intron 12, CBL Q367R – subclonal, CDKN2A/B loss, MYST3 amp	0%	0%	stable	3
3	KRAS amplification, MET amplification, CDK6 amplification, HGF amplification – equivocal, CDKN2A/B loss, MYST3 amplification – equivocal, PIK3CG amplification, RUNX1 duplication exon 6, TP53 Y220C	N/D	N/D	stable	7
4	CREBBP Y1450C, MLL2 Q1949*, PIK3R1 Q329*	0%	0%	stable	3
5	FGF19 amplification, FGF4 amplification, FGF23 amplification, FGF3 amplification, FGF6 amplification, CCND1 amplification, KRAS amplification, CDKN2A/B loss, MYC duplication exons 2-3, TP53 E204*, CHD2 D213N, CREBBP R1392*, EMSY amplification, KDM5A amplification.	N/D	N/D	N/D	N/D
6	N/D	N/D	N/D	N/D	N/D
7	EGFR exon 20 insertion (N771>HH), TP53 E258*	0%	0%	Unknown	Unknown
8	CCNE1 amplification, TP53 G245S	1%	0%	Intermediate	10
9	CCND1 amplification, CDK4 amplification – equivocal, KRAS G12V, FGF19 amplification, FGF4 amplification, TP53 R342*,	N/D	N/D	N/D	N/D

	BCL2L2 amplification – equivocal, CDK8 amplification, FGF3 amplification				
10†	N/D	N/D	N/D	N/D	N/D
11‡	N/D	<1%	1%	stable	3.7 m/MB (54th percentile)
12§	N/D	<1%	<1%	stable	1.1 m/MB (13th percentile)

Abbreviations: Amplification – equivocal, the assay data provide some, but not unambiguous, evidence that the copy number of a gene exceeds the threshold for identifying copy number amplification. APC, APC regulator of WNT signaling pathway; BCL2L2, BCL2 like 2; CCND1, cyclin D1; CCNE1, cyclin E1; CDKN2A/B, cyclin dependent kinase inhibitor 2A/B; CDK4, cyclin dependent kinase 4; CDK6, cyclin dependent kinase 6; CDK8, cyclin dependent kinase 8; CHD2, chromodomain helicase DNA binding protein 2; CREBBP, CREB binding protein; EGFR, epidermal growth factor receptor; EMSY, BRCA2 Interacting transcriptional repressor EMSY; FGF3, fibroblast growth factor 3; FGF4, fibroblast growth factor 4; FGF6, fibroblast growth factor 6; FGF19, fibroblast growth factor 19; FGF23, fibroblast growth factor 23; fs, frame shift; HGF, hepatocyte growth factor; KDM5A, lysine demethylase 5A; KRAS, kirsten rat sarcoma viral proto-oncogene; MET, MET receptor tyrosine kinase; MSI, microsatellite Instability; MYC, MYC proto-oncogene; MYST3, lysine acetyltransferase 6A; m/MB, mutations per megabase; N/D, not done; PD-L1, programmed death ligand 1; PIK3CA, phosphatidylinositol-4,5-bisphosphate 3-kinase catalytic subunit alpha; PIK3CG, phosphatidylinositol-4,5-bisphosphate 3-kinase catalytic subunit gamma; PIK3R1, phosphoinositide-3-kinase regulatory subunit 1; RB1, RB transcriptional corepressor 1; RUNX1, RUNX family transcription factor 1; subclonal, the analytical methodology has identified as being present in <10% of the assayed tumor DNA; TMB, tumor mutational burden; TP53, tumor protein P53.

†Pt10- Tissue collected immediately after completion of treatment on the BBI608 protocol demonstrated FGFR2 (fibroblast growth factor receptor 2) C382R.

‡Pt11- Tissue collected 2.5 years after completion of treatment on the BBI608 protocol demonstrated MYB (MYB proto-oncogene) chromosomal rearrangement, BCOR (BCL-6 transcriptional corepressor) P698fs, and KDM6A (lysine demethylase 6A) R1415*.

§Pt12- Tissue collected 2 years after completion of treatment on the BBI608 protocol demonstrated BRAF (B-Raf proto-oncogene, serine/threonine kinase) V600 duplication, MYB – NFIB (nuclear factor I B) chromosomal rearrangement, and KMT2D (lysine Methyltransferase 2D) Q2696*.

Table S3. Clinical consequences of all adverse events by patient

Pt ID	AE	Grade	Attribution/ Relation to BBI608	Attribution/ Relation to Immunotherapy	AE Onset (days from CD1)	Dose Delay	Resolved	Time to AE Resolution. days	Action Taken
1	Nausea/vomiting	1	Possible	Possible	C1D1 (0)	No	No	Ongoing	None
	Diarrhea	1	Possible	Possible	C1D1 (0)	No	No	Ongoing	None
	Colitis	3	Probable	Unrelated	C1D5 (4)	Yes	Yes	2	BBI608 held
	Gait instability	3	Unrelated	Unrelated (brain metastases)	C1D5 (4)	Yes	Yes	2	BBI608 held
2	Diarrhea	3	Possible	Unrelated	C1D3 (2)	Yes	Yes	4	BBI608 held
	Nausea	2	Possible	Unrelated	C1D3 (2)	No	Yes	4	None
	Dehydration	2	Possible	Unrelated	C1D3 (2)	Yes	Yes	4	BBI608 held
	Dyspnea	3	Unrelated	Unrelated	C1D21 (20)	Yes	Yes	1	BBI608 held
	Intermittent abdominal pain	2	Possible	Unrelated	C1D3 (2)	No	Yes	4	None
	General weakness	1	Unrelated	Unrelated	C1D3 (2)	No	Yes	4	None
3	Leg swelling	3	Unrelated	Unrelated	C1D13 (12)	Yes	Yes	3	Drug held
	Hyponatremia	3	Unrelated	Unrelated	C1D13 (12)	Yes	Yes	3	Drug held
	Dyspnea	3	Unrelated (disease-related)	Unrelated	C2D13 (12)	Yes	Yes	3	BBI608 held
	Hyponatremia	3	Unrelated	Unrelated	C2D13 (12)	Yes	Yes	3	BBI608 held
	Hyponatremia	3	Unrelated	Unrelated	C2D28 (27)	No	Yes	5	None
	Hyperkalemia	3	Unrelated	Unrelated	C2D28 (27)	No	Yes	5	None

	Fatigue	2	Unrelated	Unrelated	C2D21 (20)	No	No	Ongoing	None
4	Diarrhea	1	Definite	Definite	C1D2 (1)	No	No	Ongoing	None
	Nausea	1	Definite	Definite	C1D2 (1)	No	No	Ongoing	None
	Fatigue	1	Definite	Definite	C1D2 (1)	No	No	Ongoing	None
	Intermittent abdominal cramping	1	Definite	Definite	C1D2 (1)	No	No	Ongoing	None
	Lower back pain	1	Unrelated	Unrelated	C5D27 (26)	No	No	Ongoing	None
5	Hypothyroidism	2	Unrelated	Definite	C2D28 (7)	Yes	Yes	68	BBI608 held
	Pulmonary embolism	1	Unrelated	Unrelated	C3D29 (28)	No	No	Ongoing	None
	Hypertension	1	Unrelated	Unrelated	C2D8 (7)	No	No	Ongoing	None
	Cough	1	Unrelated	Unrelated	C4D1 (0)	No	No	Ongoing	None
6	Anemia	3	Unrelated	Unrelated	C1D12 (11)	No	Yes	9	None
	Fatigue	3	Unrelated	Unrelated	C1D13 (12)	No	Yes	8	None
	Abdominal pain	3	Probably related	Unrelated	C1D13 (12)	No	Yes	8	None
7	Pulmonary embolism	4	Unrelated	Unrelated	C2D19 (18)	Yes		4	Drug held
8	Dyspnea	3	Unrelated	Unrelated	C1D21 (20)	Yes		12	Drug held
	Orange urine	1	Possible	Unrelated	C3D1 (0)	No		21	None

	Anxiety	1	Unrelated	Unrelated	C1D27 (26)	No		Ongoing	None
	Hypokalemia	1	Unrelated	Unrelated	C2D23 (22)	No		Ongoing	None
	Acute Pain in Back	1	Unrelated	Unrelated	C3D21 (20)	No		Ongoing	None
9	Pneumonia	3	Unrelated	Unrelated	C2D21 (20)	Yes		6	BBI608 held
	Dry itchy skin	1	Possible	Possible	C4D26 (25)	No		Ongoing	None
10	Pain right side of face and jaw	3	Unrelated (disease-related)	Unrelated	C2D22 (21)	No		1	None
	Nausea	1	Unrelated	Unrelated	C1D2 (1)	No		Ongoing	None
	Trismus	1	Unrelated	Unrelated	C3D15 (14)	No		Ongoing	None
	Constipation	1	Unrelated	Unrelated	C1D2 (1)	No		Ongoing	None
	Hemoptysis	3	Unrelated	Unrelated	C8D4 (3)	Yes		2	BBI608 held
	Bronchitis	3	Unrelated	Unrelated	C8D9 (8)	Yes		2	BBI608 held
11	Colitis	3	Probable	Unrelated	C1D3 (2)	Yes		4	BBI608 held
	Diarrhea	3	Probable	Unrelated	C1D3 (2)	Yes		4	BBI608 held
	Elevated TSH	1	Unrelated	Definite	C3D1 (0)	No		Ongoing	None
	Elevated Alkaline phosphatase	1	Possible	Possible	C3D1 (0)	No		Ongoing	None
	Elevated ALT	1	Possible	Possible	C6D1 (0)	No		Ongoing	None
12	Xerodermia	1	Unrelated	Unrelated	C3D1 (0)	No		Ongoing	None
	Abdominal cramps	1	Definite	Definite	C3D1 (0)	No		Ongoing	None

	Diarrhea	1	Definite	Definite	C1D2 (1)	No		Ongoing	None
	Cancer-related pain	1	Unrelated	Unrelated	C3D28 (27)	No		Ongoing	None
	Urinary tract infection	1	Unrelated	Unrelated	C3D28 (27)	No		Ongoing	Concomitant medication
	Intermittent anemia	1	Unrelated	Unrelated	C2D15 (14)	No		Ongoing	None
	Non-productive cough	1	Unrelated	Unrelated	C5D1 (0)	No		Ongoing	Concomitant medication

Abbreviations: C, cycle; D, day

Table S4. Comparison of published data with our data on patients who were treated with other agents that may also inhibit STAT3

**Clinical trial**	**Our study**	**Phase I study of BBI608 in Japanese patients (PMID: 32236642)**	**OPB-111077 (STAT3 and OXPHOS inhibitor), phase I (PMID: 29511132)**		**OPB-51602, phase I (PMID: 25609248)**	**OPB-31121, phase I (PMID: 25715763)**	**OPB-111077, phase I (PMID: 29898591)**	**AZD9150, phase I (PMID: 26582900)**	**STAT3 antisense oligonucleotide AZD9150, phase I/Ib (PMID: 30446007)**		**OPB-51602, phase I (PMID: 25912076)**
Indications/ Diagnosis	Advanced metastatic cancer, any tumor type	Advanced solid tumors	Advanced cancer		Solid tumors refractory to standard therapy	Solid tumors refractory to standard therapy	Advanced Hepatocellular Carcinoma	Advanced lymphoma and solid tumor	Relapsed or treatment refractory lymphoma		Relapsed or refractory hematological malignancies
Dose	BBI608 (240-480 mg PO BID) with Pembrolizumab (2 mg/kg), q. 3 wks; Nivolumab (3 mg/kg), q. 4 wks; or Ipilimumab (3 mg/kg), q. 3 wks	<u>mg/day</u><br>480 (Cohort 1)<br>960 (Cohort 2)<br>1440 (Cohort 3)	Stage 1 (dose escalation): 100-400mg; Stage 2 (dose expansion): 250mg		2, 4, and 5 mg	100 mg (n=4), 200 mg (n=3), 400 mg (n=3), 600 mg (n=7), and 800 mg (n=8)	Continuous dosing (50 to 400 mg QD) and intermittent dosing (4-days on/3-days off administration 300 to 900 mg)	2 mg/kg (n=4)<br>3 mg/kg (n=12)<br>4 mg/kg (n=9)	2 mg/kg (n=10)<br>3 mg/kg (n=20)		1, 2, 3, 4, and 6 mg
No. of patients											
Enrolled	16	14	Stage 1, n=18<br>Stage 2, n=127		51	25	33 (continuous dosing, n=19; intermittent dosing, n=14)	25	33		20
Treated	12	14	Stage 1, n=18<br>Stage 2, n=127		51	25	33 (continuous dosing, n=19; intermittent dosing, n=14)	25	30 (DLBCL, n=27; FL, n=2; HL, n=1)		20
Age, yrs<br>Median (range)	54 (31-78)	61.5 (45–76)	<u>Mean</u><br>Stage 1: 64.4<br>Stage 2: 60.9		60 (28-81)	53 (19-76)	54 (23-69)	63 (27-85)	2mg/kg: 69 (23-83)<br>3mg/kg: 65 (22-81)		64 (49–74)
ECOG											
			Stage 1	Stage 2					2mg/kg	3mg/kg	
0	1	13	7	35	16	6	10	25 (ECOG≤2)	4	3	20 (ECOG 0-1)
1	11	1	11	86	35	18	23		6	15	
2	0		0	6	0	1	0		0	2	

Prior therapies									
Median (range)						1 (1-7)	5 (2-11)	4 (1–9)	3.5 (1–9)
0-2	5	3		13	7	25			
≥3	6	11		38	18	8			
			Prior therapy<br><br>Stage 1:<br>surgery, n=15<br>radiotherapy, n=9<br>chemotherapy/hormone, n=16<br>Stage 2:<br>surgery, n=107<br>radiotherapy, n=69<br>chemotherapy/hormone, n=127						
Adverse events									
**Treatment-related**									
Evaluable	N=12	N=14	N=127 (Stage 2)	N=51	N=25	N=33	N=25	N=30	N=20
Most common (≥20%)	Diarrhea (n=5, 41.7%)<br>Nausea (n=4, 33.3%)	Diarrhea (n=9, 64.3%)<br>Nausea (n=4, 28.6%)<br>Vomiting (n=3, 21.4%)<br>Anorexia (n=3, 21.4%)	Nausea (n=87, 68.5%)<br>Fatigue (n=76, 59.8%)<br>Vomiting (n=61, 48.0%)<br>Constipation (n=47, 37.0%)<br>Dizziness (n=40, 31.5%)<br>Anorexia (n=30, 23.6%)	Vomiting (n=39, 76.4 %)<br>Nausea (n=34, 66.7%)<br>Fatigue (n=25, 49.0%)<br>Diarrhea (n=23, 45.0%)<br>Neuropathy (n=19, 37.2%)<br>Anorexia (n=18, 35.3%)	Nausea (n=21, 84.0%)<br>Vomiting (n=20, 80.0%)<br>Diarrhea (n=18, 72.0%)	Decreased appetite (n=12, 36.4%)<br>Dizziness (n=14, 42.4%)<br>Fatigue (n=14, 42.4%)<br>Headache (n=9, 27.3%)<br>Nausea (n=28, 84.8%)<br>Vomiting (n=16, 48.5%)	ALT increased (n=11, 44.0%)<br>AST increased (n=11, 44.0%)<br>Thrombocytopenia (n=10, 40.0%)<br>Anemia (n=5, 20.0%)	ALT, AST elevation (n=12, 40.0%); Fatigue (n=11, 36.7%); Thrombocytopenia (n=9, 30.0%);<br>nausea (n=6, 20.0%)	Nausea (n=11, 55.0%)<br>Peripheral sensory neuropathy (n=9, 45.0%)<br>Diarrhea (n=8, 40.0%)<br>Decreased appetite (n=7, 35.0%)<br>Anemia (n=6, 30.0%)<br>Malaise (n=5, 25.0%)<br>Vomiting (n=5, 25.0%)<br>Neutropenia (n=5, 25.0%)<br>Thrombocytopenia (n=5, 25.0%)<br>Fatigue (n=4, 20.0%)
Grade ≥3 AEs									

	Abdominal pain (n=1, 8.3%)<br>Colitis (n=2, 16.7%)<br>Diarrhea (n=2, 16.7%)	Diarrhea (n=1, 7.1%)	AST increased (n=8, 6.3%)<br>Anemia n=6<br>GGT increased (n=7, 5.5%)<br>Nausea (n=5, 3.9%)<br>Fatigue (n=4, 3.1%)<br>Vomiting (n=4, 3.1%)<br>Constipation (n=3, 2.4%)<br>Dyspnea (n=2, 1.6%)<br>Hyponatremia (n=9, 7.1%)<br>Abdominal pain (n=1, 0.8%)<br>Dizziness (n=1, 0.8%)<br>Diarrhea (n=1, 0.8%)<br>Right ventricular dysfunction (death, SAE, n=1, 0.8%)	Electrolyte disturbance (n=4, 7.8%)<br>Neuropathy (n=4, 7.8%)<br>Infections (n=2, 3.9%)<br>Vomiting (n=1, 2.0%)<br>Diarrhea (n=1, 2.0%)<br>Dehydration (n=1, 2.0%)<br>Metabolic disturbance (n=1, 2.0%)<br>Pneumonitis (n=1, 2.0%)<br>Neutropenia (n=1, 2.0%)	Vomiting (n=2, 8.0%)<br>Diarrhea (n=1, 4.0%)	Thrombocytopenia (n=2, 6.1%)<br>Abdominal pain (n=1, 3.0%)<br>Dizziness (n=1, 3.0%)<br>Fatigue (n=1, 3.0%)	N/R	Thrombocytopenia (n=5, 16.7%)<br>ALT elevation (n=2, 6.7%)<br>AST elevation (n=1, 3.3%)<br>Fatigue (n=2, 6.7%)<br>Nausea (n=1, 3.3%)<br>Anemia (n=1, 3.3%)<br>Dysphagia (n=1, 3.3%)<br>Hypercalcemia (n=1, 3.3%),<br>Hypokalemia (n=1, 3.3%),<br>Hyponatremia (n=1, 3.3%), Neutropenia (n=3, 10.0%), Asthenia (n=1, 3.3%)	Diarrhea (n=1, 5.0%)<br>Anemia (n=1, 5.0%)<br>Neutropenia (n=4, 20.0%)<br>Thrombocytopenia (n=2, 10.0%)
Response									
Evaluable	n=12	n=14	n= 15 (stage 1), n= 93 (stage 2)	n=37	n=18	n=31	n=25	n= 30	
	SD, n= 6 (50.0%)	SD, n=2 (14.3%)	Stage 1:<br>CR, n=0 (0.0%)<br>PR, n= 0 (0.0%)<br>SD, n=10 (66.7%)<br>Stage 2:<br>CR, n=0 (0.0%)<br>PR, n= 0 (0.0%)<br>SD, n=30 (32.2%)	PR, n=2 (5.4%)	SD, n=8 (44.4%)	SD, n=13 (41.9%)	PR or SD, n=11 (44.0%)	CR, n=2 (6.7%)<br>SD, n=1 (3.3%)	N/R
PFS<br>Median, months (95% CI)	2.73 (0.66-8.05)	Cohort 1, 1.87<br>Cohort 2, 1.76<br>Cohort 3, 1.45	N/R	N/R	N/R	Overall:<br>1.4 (0.8-2.8)<br>continuous dosing: 1.6 (0.8 to 4.2)	N/R	N/R	N/R

						intermittent dosing: 0.9 (0.7-2.8)			
OS Median, months (95% CI,)	7.56 (1.22 months-NR)	Cohort 1, 8.28<br>Cohort 2, 5.03<br>Cohort 3, 5.68	N/R	N/R	N/R	Median, NR; 6-month, 44.4%; 12-month, 11.1%	N/R	N/R	N/R

Abbreviations: Alanine aminotransferase (ALT); Aspartate aminotransferase (AST); BID, twice daily; CR, complete response; DLBCL, diffuse large B-cell lymphoma; FL, follicular lymphoma; HL, Hodgkin lymphoma; NR, not reached; N/R, not reported; OS, overall survival; PD, partial response; PFS, progress free survival; PR, partial response; PO, *per os* (orally); q., every; SD, stable disease; wks, weeks.

Table S5. Comparison of published data with our data on patients who were treated with immunotherapy.

**Clinical trial**	**Current study**	**Pembrolizumab, phase I (PMID: 25977344)**	**Pembrolizumab + BBI608, phase I/II (PMID: 32694160)**	**Nivolumab, phase I (PMID: 20516446)**	**Ipilimumab https://www.accessdata.fda.gov/drugsatfda_docs/label/2020/125377s115lbl.pdf**	**Ipilimumab, phase I (PMID: 26534966)**
Indications/ Diagnosis	Advanced metastatic cancer, any tumor type	Advanced solid tumors	Metastatic colorectal cancer	Refractory solid tumors		Pediatric Patients with Advanced Solid Tumors
Dose	BBI608 (240-480 mg PO BID) Pembrolizumab (2 mg/kg), every 3 weeks Nivolumab (3 mg/kg), every 4 weeks Ipilimumab (3 mg/kg), every 3 weeks	Pembrolizumab 1, 2, 3 or 10 mg/kg q. 2 wks (Part A) 10 mg/kg q. 2 wks (Part A-1) 2 or 10 mg/kg q. 3 wks (Part A-2)	BBI608 (240-480 mg PO BID) Pembrolizumab 200 mg q. 3 wks	Nivolumab 0.3, 1, 3, or 10 mg/kg q. 3 wks	Ipilimumab (3 mg/kg)	Dose escalation cohorts: 1, 3, 5, or 10 mg/m^2 q. 3 wks
No. of patients						
Enrolled	16	32	55	39		33
Treated	12	30	50	39	511	33
Age at the time of treatment in yrs Median, range	54 (31-78)	66.5 (33-87)	Cohort A (MSI-H), 53 (30-77) Cohort B (MSS), 63 (25-79)	62 (42-84)		13.4 (2-21)
ECOG						
0	1	10	44	13		N/R
1	11	20	6	26		
Prior therapies						
0-2	5	15	18	Median (range), 4 (1-13)		Chemotherapy, 0-2, n=9; ≥3, n=14 Radiation, n=17 Biologic (IL2, IFNγ or adoptive T-cell therapy), n=11
≥3	6	15	32			
Adverse events						
**Treatment-related**						
Most common	Diarrhea (n=5, 41.7%) Nausea (n=4, 33.3%)	Fatigue (n=10, 33%) Nausea (n=7, 23%)	Diarrhea (n=43, 86%) Nausea (n=15, 30%) Decreased appetite (n=13, 26%)	Decreased CD4+ lymphocyte counts (n=14, 35.9%) Lymphopenia (n=10, 25.6%)	Rash (n=76, 14.5%) Diarrhea/colitis (n= 62, 12.1%) Hepatitis (n=21, 4%)	Colitis/diarrhea (n=4, 12%) Rash (n=4, 12%) Transaminitis, endocrinopathies, other irAE (n=3, each, 9.1%)

			Fever (n=10, 20%)	Fatigue and musculoskeletal events (n=6 each, 15.4%)		
Grade ≥3 AEs	Diarrhea (n=2, 16.7%)<br>Colitis (n=2, 16.7%)<br>Abdominal pain (n=1, 8.3%)	None	Diarrhea (n=2, 4%)<br>Decreased appetite (n=3, 6%)<br>Fever, nausea, rash, abdominal pain, anemia, hypokalemia, impaired hearing, colitis,<br>serum amylase increased, alkaline phosphatase increased (n=1 each, 2%)	CD4 decrease (n=7, 17.9%)<br>Anemia, lymphopenia, hypocalcemia,<br>ascites, colitis, fatigue, musculoskeletal disorder (n=1 each, 2.6%)		Colitis/diarrhea (n=3, 9.1%)<br>Transaminitis (n=2, 6.1%)<br>Endocrinopathy (n=1, 3%)<br>Other irAE (n=3, 9.1%)
Response						
Evaluable	N=12	N=30	N=50	N=39	N/R	N=33
	SD, n= 6 (50%)<br>PD, n=5 (41.7%)<br>Clinical Progression, n=1 (8.3%)	CR, n= 2 (6.7%)<br>PR, n=3 (10%)<br>SD, n=15 (50%)<br>PD, n=6 (20%)<br>No tumor assessment, n=4 (13.3%)	Cohort A (N=10), CR, n=1; PR, n=4; SD, n=4, PD, n=1<br>Cohort B (N=40), PR, n=4; SD, n=14, PD, n=21, NE, n=1	CR, n=1 (2.5%)<br>PR, n=2 (5.1%)<br>MXR, n=2 (5.1%)		SD, n=4 (12.1%)
PFS<br>Median, months (95% CI)	2.73 (0.66-8.05)	N/R	NR (95% CI, 1.4 months–NR) (Cohort A)<br>1.6 months (95% CI, 1.4–2.1) (Cohort B)	N/R	N/R	N/R
OS<br>Median, months (95% CI)	7.56 (1.22 months-not reached)	N/R	NR (95% CI, 3.3 months–NR) (Cohort A)<br>7.3 months (95% CI, 5.3–11.8 months) (Cohort B)	N/R	N/R	N/R

Abbreviations: BID, twice daily; CR, complete response; MSI-H, microsatellite instability high; MSS, microsatellite stable; MXR, mixed response; NE, not evaluated; NR, not reached; N/R, not reported; PD, partial response; PR, partial response; PO, *per os* (orally); SD, stable disease

Supplemental figures

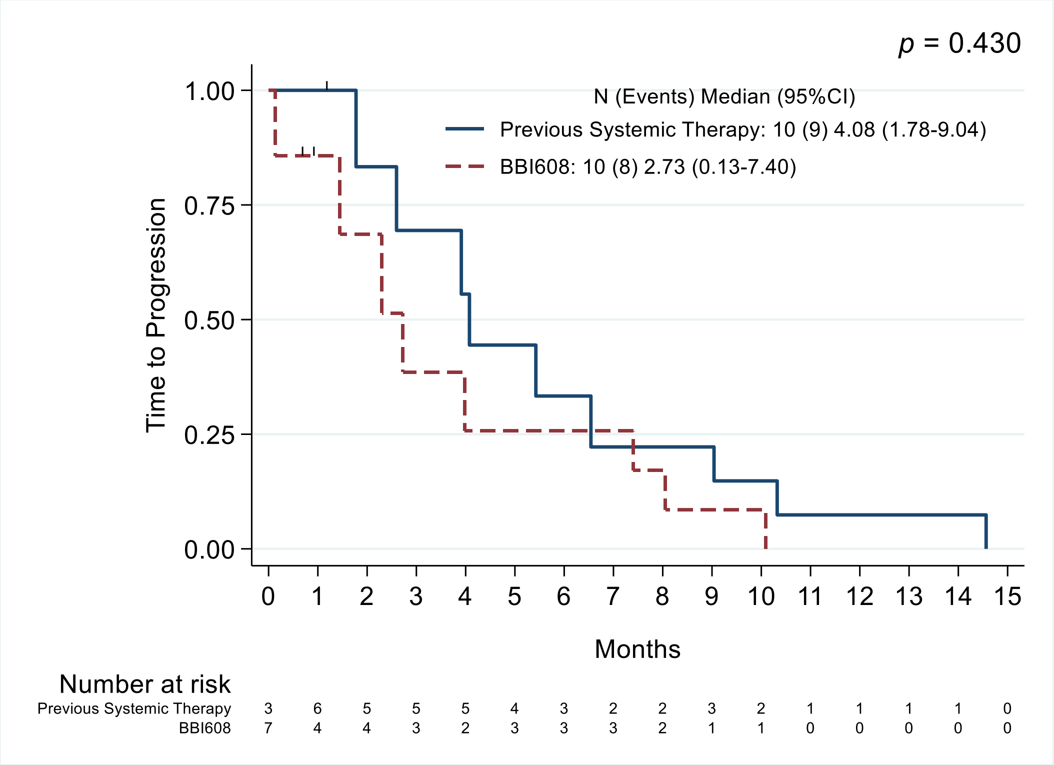

Figure S1. PFS of 10 patients treated with BBI608 and immunotherapy versus prior systemic therapy. Two of 12 patients received BBI608 and immunotherapy but did not have prior systemic therapy, and therefore they were not included in the analysis.

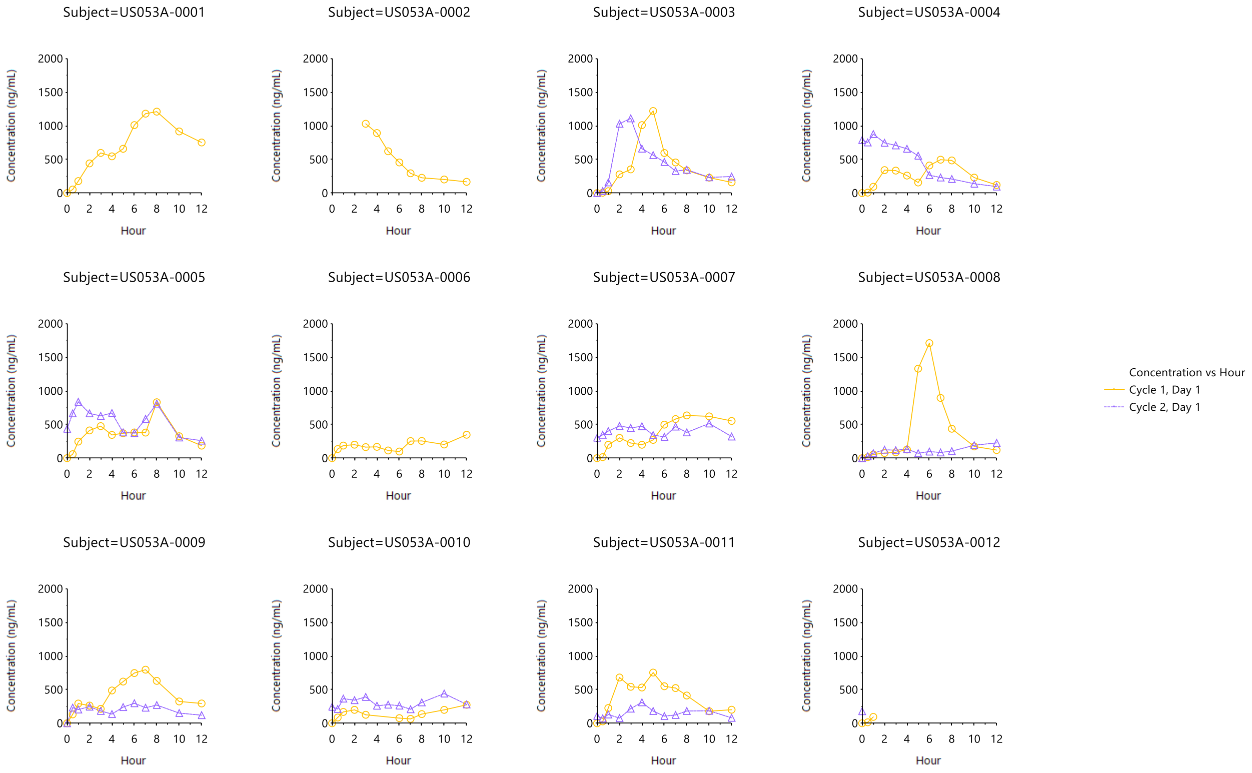

Figure S2. BBI608-201CIT Individual PK Profiles (linear). Below limit of quantification samples were set to “0” in figures.

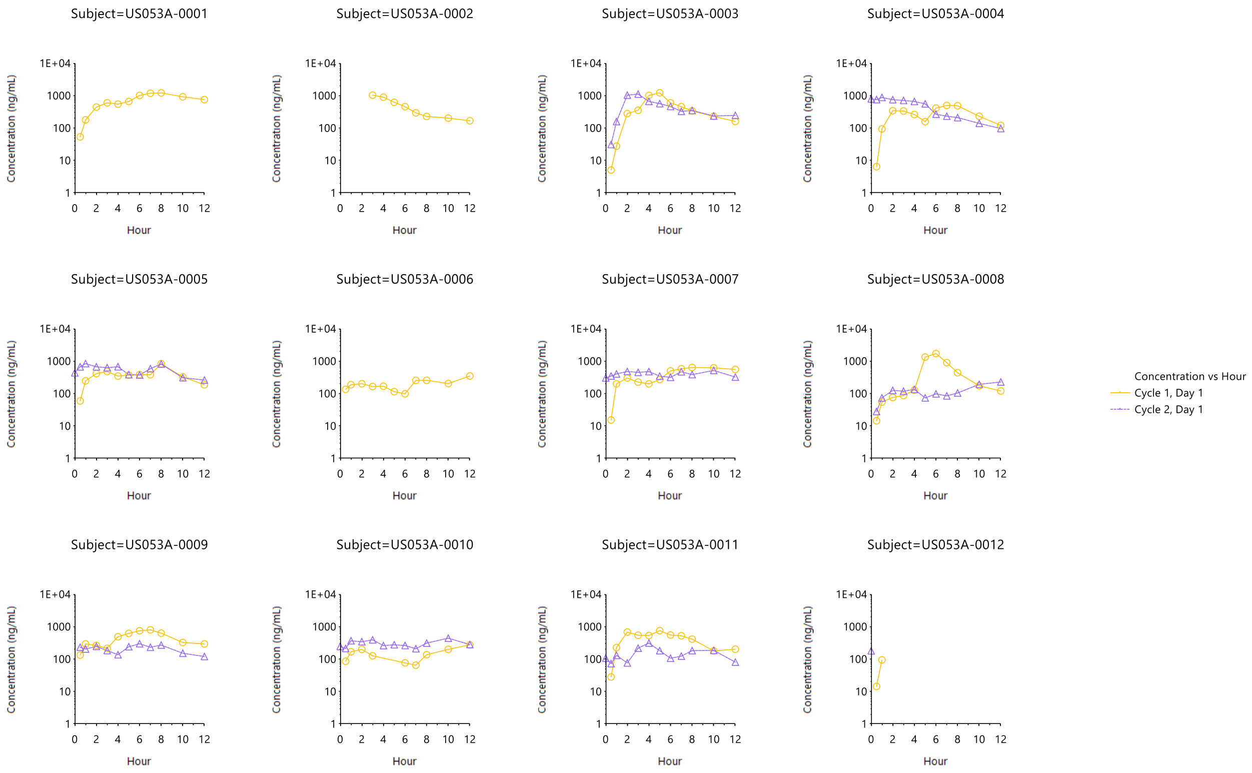

Figure S3. BBI608-201CIT Individual PK Profiles (semi-log). Below limit of quantification samples were set to "0" in figures.

Cycle=Cycle 1, Day=Day 1

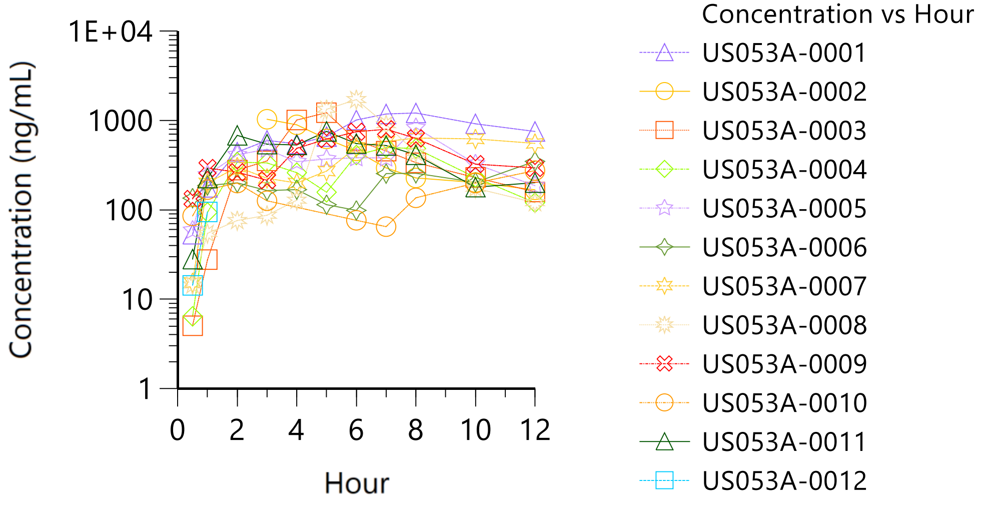

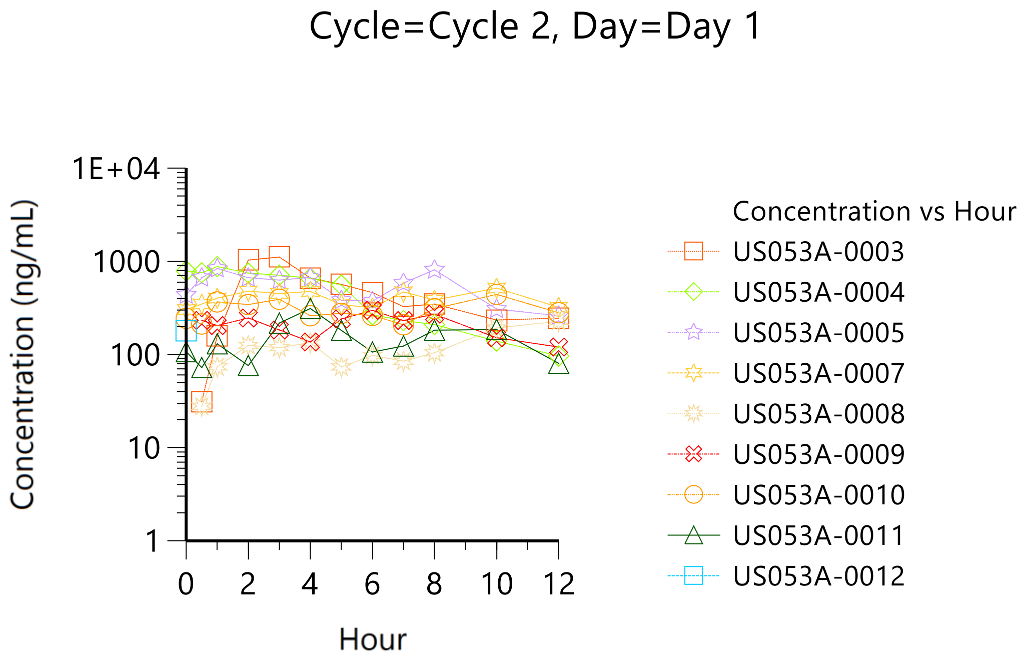

Figure S4. BBI608-201CIT PK Profiles (semi-log). Below limit of quantification samples were set to “0” in figures.

Cycle=Cycle 1, Day=Day 1

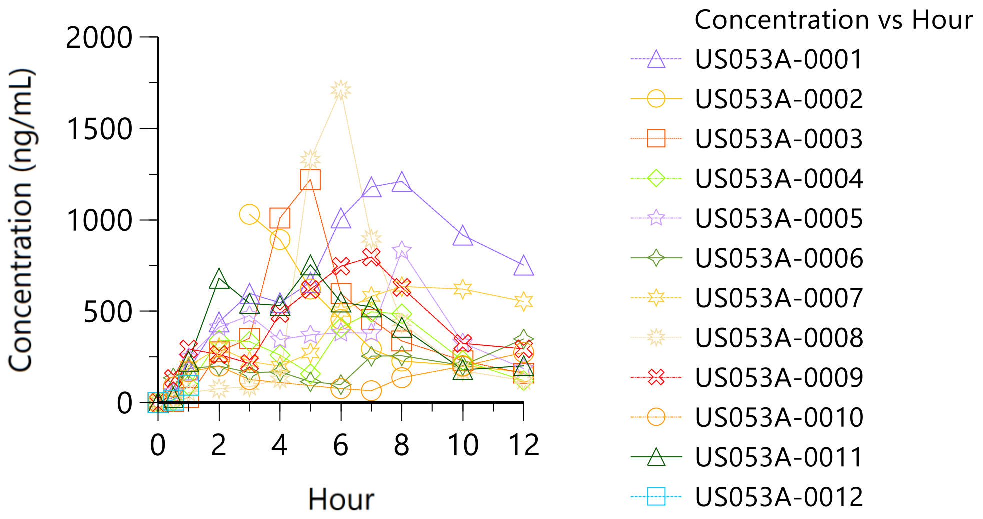

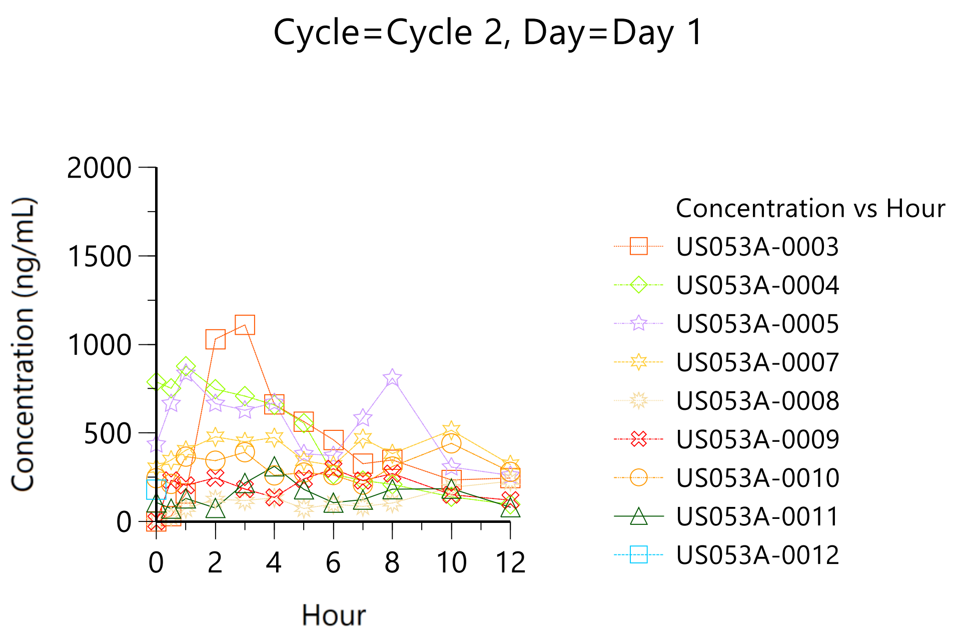

Figure S5. BBI608-201CIT PK Profiles (linear). Below limit of quantification samples were set to "0" in figures.

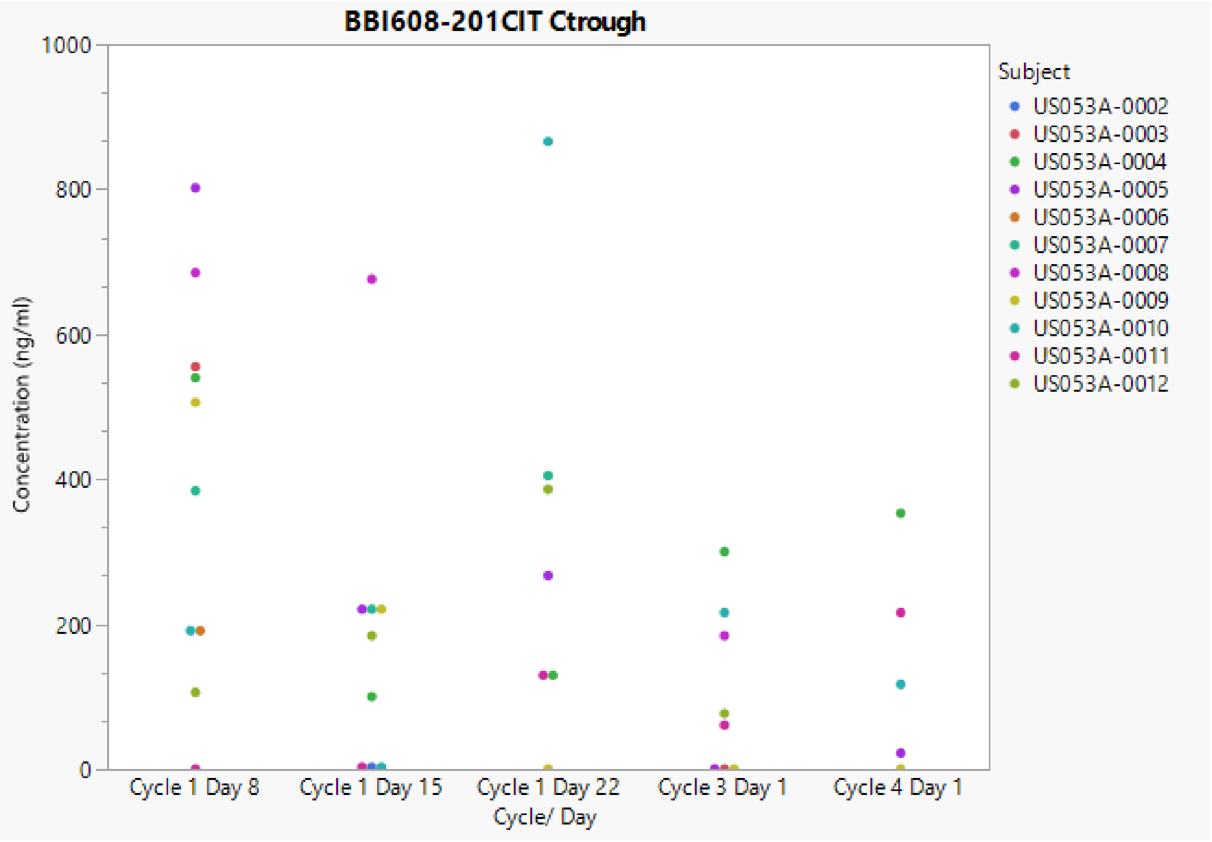

Figure S6. Trough concentration levels of BBI608 of all patients. Below limit of quantification samples were set to "0".
